# How to Conduct a Bayesian Network Meta-Analysis

**DOI:** 10.3389/fvets.2020.00271

**Published:** 2020-05-19

**Authors:** Dapeng Hu, Annette M. O'Connor, Chong Wang, Jan M. Sargeant, Charlotte B. Winder

**Affiliations:** ^1^Department of Statistics, Iowa State University, Ames, IA, United States; ^2^Department of Large Animal Clinical Sciences, College of Veterinary Medicine, Michigan State University, East Lansing, MI, United States; ^3^Department of Veterinary Diagnostic and Production Animal Medicine, 2203 Lloyd Veterinary Medical Center, Iowa State University, Ames, IA, United States; ^4^Department of Population Medicine, University of Guelph, Guelph, ON, Canada

**Keywords:** network meta-analysis, Bayesian, systematic review, tutorial, veterinary science

## Abstract

Network meta-analysis is a general approach to integrate the results of multiple studies in which multiple treatments are compared, often in a pairwise manner. In this tutorial, we illustrate the procedures for conducting a network meta-analysis for binary outcomes data in the Bayesian framework using example data. Our goal is to describe the workflow of such an analysis and to explain how to generate informative results such as ranking plots and treatment risk posterior distribution plots. The R code used to conduct a network meta-analysis in the Bayesian setting is provided at GitHub.

## 1. Introduction

Meta-analysis is a quantitative method commonly used to combine the results of multiple studies in the medical and veterinary sciences. There are several common types of meta-analysis. A pairwise meta-analysis compares two treatments across multiple studies, whereas a network meta-analysis involves the simultaneous synthesis of multiple studies to create pairwise comparisons of more than two treatments. A third type of meta-analysis is multivariate meta-analysis, which is far less common than the other two types (1, 2). Regardless of the type, meta-analyses can be conducted using study-level summary data, which are usually reported in the literature. In the human health sciences, it is also possible to perform meta-analyses using data from individual patients, but meta-analysis using individual-level data is very rare in veterinary science (3).

In this tutorial, we focus on network meta-analysis, which is becoming increasingly common in both human health and the veterinary sciences (4–10). Although frequently used as a synonym for network meta-analysis, a mixed treatment comparisons meta-analysis is a type of network meta-analysis that can be described as a “*A statistical approach used to analyze a network of evidence with more than two interventions which are being compared indirectly, and at least one pair of interventions compared both directly and indirectly”* (1). Direct comparisons of interventions are obtained from trials or observational studies that include both interventions and compare them directly. Indirect comparisons of interventions, on the other hand, are made based on multiple trials that each included one, but not both, of the interventions of interest and therefore did not compare the interventions directly as part of the original study. In general, network meta-analysis offers the advantage of enabling the combined assessment of more than two treatments. A network meta-analysis that includes the mixed treatment comparisons “component” has the additional feature of enabling a formal statistical estimation of indirect treatment comparisons that might not be available in the literature (4, 7). Most network meta-analyses include a mixed treatment comparisons component, so we use the term network meta-analysis to refer to mixed treatment comparisons meta-analyses throughout this manuscript. There are some R (11) packages available for conducting Bayesian network meta-analysis such as *gemtc* (12) and *BUGSnet* (13). The output given by *gemtc* is limited. For example, *gemtc* does not have the option to report the summary effect as either the relative risk or absolute risk. Further, the output is not available in a table format. While *BUGSnet* is limited to analyzing arm-level data which could be a limitation for veterinary data which is often reported at the contrast level.

### 1.1. Rationale

Currently, only a few systematic reviews in veterinary science have employed network meta-analysis. However, if the trend in the human health sciences is indicative of what will occur in veterinary science, we can expect to see more network meta-analyses of veterinary studies in the future. For example, in 2010, a PubMed search with the terms “network meta-analysis” OR “mixed treatment comparison” yielded 10 citations, whereas by 2018, the same search returned 618 citations. The rise in the use of network meta-analysis is a function of the value that such an analysis provides to the decision-making community. Instead of limiting comparisons to those that are made across just two interventions and published in the literature, as is the case for pairwise meta-analysis, network meta-analysis allows the simultaneous comparison of multiple treatments, including comparisons that are not directly available in the literature. For many clinical decisions in veterinary medicine, there are multiple interventions that could be used to prevent or treat a specific disease or condition. Therefore, decision-makers are interested in the comparative efficacy of all the options rather than just pairwise comparisons. To illustrate the limitations of pairwise meta-analysis, we can use the choice of which antibiotic to use to treat bovine respiratory disease as an example. The vast majority of publicly available trials involving antibiotics for bovine respiratory disease were conducted in order to register and license a particular product. In those types of trials, the antibiotic of interest is typically compared with a placebo to demonstrate that the antibiotic has a significant beneficial effect. Veterinarians are actually interested in the comparative efficacy of all the available antibiotics, but for a variety of reasons (e.g., economic, marketing, and regulatory), few head-to-head comparisons of antibiotics are available. A network meta-analysis can fill that information gap for veterinarians by providing head-to-head estimates of the comparative efficacy of antibiotics, even though those comparisons are not available in the literature.

### 1.2. Objectives

Our objective is to provide a tutorial illustrating how to conduct a network meta-analysis of study-level results from multiple sources. Network meta-analysis can be conducted using a frequentist approach or a Bayesian approach. We focus on the Bayesian approach for three reasons:

- First, Bayesian approaches to network meta-analysis are currently more common than frequentist approaches (14–16).
- Second, the learning curve for the Bayesian approach is steeper than that for the frequentist approach. There are several standard packages that can be used to conduct a frequentist analysis, and the examples provided with the packages are usually sufficient to enable the analysis to be conducted (17, 18). Therefore a tutorial for the Bayesian approach fills a larger gap.
- Third, the Bayesian approach allows for many outputs that enhance understanding of the data. For example, the point estimate, as well as the posterior distribution of the absolute risk of each treatment can be obtained from the results of the Bayesian approach. Therefore, a tutorial focused on the Bayesian approach to network meta-analysis has greater utility.

### 1.3. Target Audience

We describe the step-wise workflow of a network meta-analysis, and we provide R, JAGS (19) and BUGS (20) code for end-users interested in troubleshooting or optimizing their own analyses (see Appendix for link). It is not our intention to teach the statistical foundations of network meta-analysis. We believe that this tutorial will fill a gap between papers that explain the underlying statistical methodology and the “black box” tutorials that typically come with statistical packages. Our tutorial is intended for readers interested in understanding the software-coding and data-management processes that underlie a network meta-analysis. It is our hope that by using our tutorial, a reader would be able to find errors in his or her own network meta-analysis or modify existing code to produce a new output. We assume that the reader is familiar with pairwise meta-analysis [see the companion paper in the frontiers series (21) and the paper about synthesizing data from intervention studies using meta-analysis (22) for more details].

## 2. Organization

The tutorial is organized in three parts. First, we provide a basic introduction to Bayesian network meta-analysis and the concepts in the underlying model. Second, we discuss how to conduct the analysis, with a focus on the software processes involved. Third (in the Appendix), we provide actual code that can be used to conduct a Bayesian network meta-analysis. The Appendix contains detailed instructions on how to run the R code that will perform the analysis and produce the desired outputs. The code includes R and jags scripts for executing a network meta-analysis in an R project, which contains several scripts that the reader can run to better understand the processes associated with conducting the analysis and obtaining the output. Not all readers will want to delve into the mechanisms of the Appendix code. For readers who want to conduct a network meta-analysis but are not interested in the mechanics of coding the analysis, we suggest that they read the first two parts of the tutorial and then use an R package that includes functions for running a network meta-analysis, such as *gemtc*.

## 3. The Basics of Network Meta-Analysis

### 3.1. Arm-Level Data and Contrast-Level Data

The first part of a network meta-analysis is data extraction from the primary sources, preferably based on a systematic review conducted using an *a priori* protocol. The data extracted from the primary sources are study-level summary data (also called aggregated data) in one of two forms: arm-level data, which report the effect measures (i.e., absolute odds or absolute risk) for each arm, or contrast-level data, which show the contrast of effects, or the effect size (23), between treatment arms (i.e., the odds ratio, relative risk, or log odds ratio). Either type of summary data can be used in a network meta-analysis using either the Bayesian or the frequentist approach (7).

It is essential, however, that the data extracted for a network meta-analysis meet the transitivity assumption, that is, that each enrolled subject in a given study would be eligible for enrollment in the other studies. For example, in a previous network meta-analysis of antibiotic treatments for bovine respiratory disease, data from studies that included antibiotic metaphylaxis were excluded, because animals that received prior antibiotic treatment would have limited eligibility for subsequent antibiotic treatments and would therefore violate the transitivity assumption (5, 6). Animals that received an antibiotic as a metaphylactic treatment would be unlikely to receive the same antibiotic as the first treatment of choice once bovine respiratory disease was diagnosed. Moreover, the effect of an antibiotic might be different if the antibiotic was previously used for metaphylactic treatment in the same animal, so the results from studies with and without metaphylaxis would not be the same. By limiting the network of eligible studies for the meta-analysis to those without metaphylaxis, the transitivity assumption would be more likely to hold.

### 3.2. The Comparative Effects Model

A key aspect of network meta-analysis is the comparative effects model. The comparative effects model forms the basis for the estimation of the relative treatment effects, which make up the main output of the network meta-analysis. A commonly used approach to network meta-analysis is to directly describe the distributions of the log odds ratio as the measures of the relative treatment effects and then to transform the log odds ratios into more interpretable metrics such as odds ratios or risk ratios. The goal of the comparative effects model is to provide a mechanism to estimate the comparative treatment effects. A critical aspect of the comparative effects model and its relation to network meta-analysis is the consistency assumption. The comparative effects model provides estimates of basic parameters in the form of log odds ratios based on comparisons between each treatment of interest and a baseline treatment. The consistency assumption allows pairwise comparisons between the treatments of interest to be estimated as functions of the basic parameters estimated in the comparative effects model. This consistency assumption is written as:

$$\begin{array}{l} d_{k_{1},k_{2}}=d_{bk_{2}}-d_{bk_{1}}, \end{array}$$

where *b* is the baseline treatment, *k*_1_ and *k*_2_ are treatments other than the baseline, and *d*_*b*_*k*__2__ is the true effect size (log odds ratio in this case) of treatment *k*_2_ compared with the baseline *b*. In lay terms, using the example of bovine respiratory disease, the consistency assumption says that we can compare the effect of oxytetracycline (*k*_2_) with that of tulathrymycin (*k*_1_) if we have comparisons of the effects of oxytetracycline (*k*_2_) and a placebo (*b*) and of tulathrymycin (*k*_1_) and a placebo (*b*).

#### 3.2.1. The Fixed Effects Model and the Random Effects Model

The first factor to consider in the comparative effects model is whether the intervention effects are fixed effects or random effects. Suppose there are *N* studies in a network, which is composed of *K* treatments. Let *b* denote the baseline treatment of the whole network, and let *b*_*i*_ denote the trial-specific baseline treatment in trial *i*. It might be the case that *b*_*i*_ ≠ *b*. In other words, the baseline treatment of the model is a placebo, because most of the studies include a placebo group, but a few studies lack a placebo arm and therefore use a different treatment as the baseline comparator. Let *y*_*i*_*b*__*i*_*k*_ be the trial-specific log odds ratio of treatment *k* compared with *b*_*i*_ in trial *i*, and let *V*_*i*_*b*__*i*_*k*_ be its within-trial variance. Assume a normal distribution for *y*_*i*_*b*__*i*_*k*_, such that

$$\begin{array}{l} y_{ib_{i}k}\sim \text{N}\left(\theta_{ib_{i}k},V_{ib_{i}k}\right). \end{array}$$

The difference between a fixed effects model and a random effects model lies in the assumptions about the nature of the between-trial variability (24). The choice of the fixed effects or random effects model depends on the interpretation of the log odds ratio (θ_*i*_*b*__*i*_*k*_) and the assumptions behind that interpretation. A fixed effects model assumes that there is one true effect size underlying the trials for each comparison. It follows that all of the differences in the observed effect sizes are due to random variation (sampling error) (25), which is akin to assuming that if all the studies were of infinite size, each would result in the same effect size. In that scenario, under the consistency assumption, the model would be:

$$\begin{array}{l} \theta_{i,b_{i}k}=d_{b_{i}k}=\left\{ \begin{array}{cc} d_{bk}, & \text{for }b_{i}=b,\\ d_{bk}-d_{bb_{i}}, & \text{for }b_{i}\neq b, \end{array}.\right. \end{array}$$

In this model, *d*_*bk*_ (*k* ∈ {1, 2, …, *K*}) are called basic parameters, whereas *d*_*b*_*i*_*k*_ (*k* ∈ {1, 2, …, *K*}, *b*_*i*_ ≠ *b*) are called functional parameters, because they are a function of the basic parameters (e.g., *d*_*b*_*i*_*k*_ = *d*_*bk*_ − *d*_*b*_*b*__*i*__). For example, consider a trial (*i* = 1) that compared treatment A with treatment B. We might designate treatment A as the baseline treatment (*b*) and treatment B as *k*. The model assumes that the log odds ratio observed in study *i* = 1 is *d*_*bk*_. Any difference between the observed log odds ratio and *d*_*bk*_ is assumed to be due to sampling error. In another trial (*i* = 2) that compared treatment B to treatment C, we might designate treatment C as the baseline treatment (*b*_*i*_). When modeling the data, we would retain treatment B as *k*. The model then assumes that the observed log odds ratio in study *i* = 2 (i.e., treatment C compared with treatment B) is given by *d*_*bk*_ - *d*_*b*_*k*__*i*__. Again, any difference between the observed log odds ratio and *d*_*b*_*k*__*i*__ is assumed to be due to sampling error in a fixed effects model.

A random effects model, on the other hand, assumes that the true effect size can differ from trial to trial, because the effect sizes in each trial are derived from a distribution of effect sizes, which is akin to saying that even if the studies were all of infinite size, there would still be different estimates of the effect size due to the distribution of effect sizes in addition to sampling error. Therefore, in a random effects model, there is an additional source of variation that needs to be accounted for, that is, the between-trial variation. The random effects model has been recommended for cases in which there is heterogeneity among the results of multiple trials (26). The common distribution of the between-trial variation is usually assumed to be a normal distribution (7), so that

$$\begin{array}{l} \theta_{i,b_{i}k}\sim \left\{ \begin{array}{cc} N\left(d_{bk},\sigma_{b_{i}k}^{2}\right), & \text{for }b_{i}=b,\\ N\left(d_{bk}-d_{bb_{i}},\sigma_{b_{i}k}^{2}\right), & \text{for }b_{i}\neq b, \end{array},\right. \end{array}$$

where $\sigma_{b_{i}k}^{2}$ is the between-trial variance. In a pairwise meta-analysis, because there is only one effect size of interest, there is inherently only one between-trial variance. By contrast, in a network meta-analysis, there are at least two, and often many more, effect sizes, because we have (*k* ∈ {1, 2, …, *K*}). It is often assumed, however, that there is still only a single between-trial variance for all the treatments, which is referred to as the homogeneous variance assumption (i.e., $\sigma_{b_{i}k}^{2}=\sigma^{2}$). In lay terms, this means that if we employ a random effects model that has three treatments and therefore two effect sizes, we assume the same $\sigma_{b_{i}k}^{2}$ for *d*_*b*_*k*__1__ and *d*_*b*_*k*__2__. Although models that allow heterogeneous between-trial variances have been proposed (4, 27), we use a random effects model with an assumption of homogeneous variance as our example in this tutorial, because such a model is consistent with our biological understanding of the types of interventions used in veterinary science.

### 3.3. Handling Multi-Arm Trials

In a pairwise meta-analysis, only one effect size is obtained from each study, which means that each effect size is independent of the others. However, in a network meta-analysis, there is the potential, and often the desire, to include multi-arm trials, which creates non-independent observations. For example, a single trial might compare treatments A, B, and C, resulting in three comparisons (A to B, B to C, and B to C). If A is the baseline treatment, then the comparisons between A and B and between A and C are basic parameters. When data from such a trial are included in a network meta-analysis, the assumption of independence is not valid and needs to be adjusted. A term to adjust for the co-variance of data from multi-arm trials must be incorporated into the comparative effects model to correctly reflect the data-generating mechanism. For a single multi-arm trial with *k*_*i*_ treatments, there are (*k*_*i*_ − 1) comparisons ${\left(y_{i,b2},y_{i,b3},\ldots ,y_{i,bk_{i}}\right)}^{T}$. The joint distribution of the comparisons is given by

$$\begin{array}{l} \left(\begin{array}{c} y_{i,b2}\\ y_{i,b3}\\ \vdots \\ y_{i,bk_{i}} \end{array}\right)\sim N_{k_{i}-1}\left(\left(\begin{array}{c} \theta_{i,b2}\\ \theta_{i,b3}\\ \vdots \\ \theta_{i,bk_{i}} \end{array}\right),\left[\begin{array}{cccc} V_{i,b2} & V_{i,b} & \cdots & V_{i,b}\\ V_{i,b} & V_{i,b3} & \cdots & V_{i,b}\\ \vdots & \vdots & \ddots & \vdots \\ V_{i,b} & V_{i,b} & \cdots & V_{i,bk_{i}} \end{array}\right]\right), \end{array}$$

where *V*_*i, b*_ is the observed variance in the baseline arm in trial *i*. The derivation of the value of the co-variance can be found elsewhere (7). For a random effects model, assuming a homogeneous between-trial variance for all trial-specific effects, the joint distribution of ${\left(\theta_{i,b2},\theta_{i,b3},\ldots ,\theta_{i,bk_{i}}\right)}^{T}$ is

$$\begin{array}{l} \left(\begin{array}{c} \theta_{i,b2}\\ \vdots \\ \theta_{i,bk_{i}} \end{array}\right)\sim N_{k_{i}-1}\left(\left(\begin{array}{c} d_{b2}\\ \vdots \\ d_{bk_{i}} \end{array}\right),\left(\begin{array}{cccc} \sigma^{2} & \sigma^{2}/2 & \ldots & \sigma^{2}/2\\ \vdots & \vdots & \ddots & \vdots \\ \sigma^{2}/2 & \sigma^{2}/2 & \cdots & \sigma^{2} \end{array}\right)\right). \end{array}$$

The reason that the off-diagonal values in the variance–covariance matrix are equal to half the diagonal values (28) (i.e., the correlation is 0.5) is that we want to keep the assumption of homogeneous between-trial variance valid. For example,

$$\begin{array}{l} \text{Var}\left(\theta_{i,23}\right)=\text{Var}\left(\theta_{i,b3}-\theta_{i,b2}\right)=\text{Var}\left(\theta_{i,b3}\right)+\text{Var}\left(\theta_{i,b2}\right)-2\text{Cov}\left(\theta_{i,b3},\theta_{i,b2}\right)\\ \;=\sigma^{2}+\sigma^{2}-2*\sigma^{2}/2=\sigma^{2}. \end{array}$$

### 3.4. Choice of Priors

So far, we have described the comparative effects model, which describes how the data were generated. The next step is to estimate the parameters of the distributions of interest, that is, the basic parameters for each treatment and the between-trial variance. For a frequentist approach, model parameters are regarded as unknown fixed population characteristics (14) and estimation could be performed using a likelihood approach. The frequentist approach does not use prior information to estimate the parameters. By contrast, the Bayesian approach to estimation calculates the posterior distribution of the parameters by using the data (likelihood) to update prior information. In the Bayesian approach, it is necessary obtain a prior distribution of the parameters, so that the prior distribution can be updated to give the posterior distribution.

**Figure 1:**
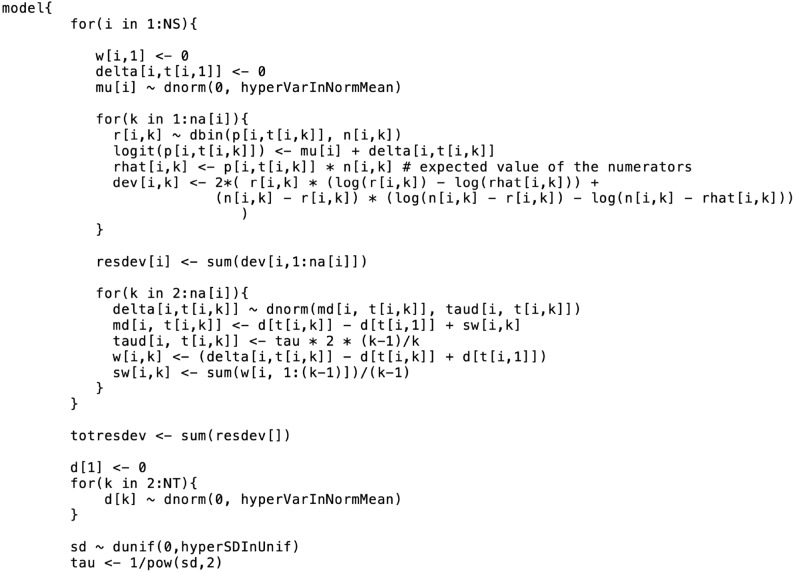
An example of the formatting of the BUGS code for the comparative model. This code was modified from code originally published elsewhere (7).

Prior distributions must be selected for the basic parameters *d*_*bk*_ (*k* ∈ {1, 2, …, *K*}) and, if a random effects model is employed, also the between-trial variance σ^2^. There is no need to select a prior for the correlation of multi-arm trials, because that correlation is constrained to 0.5 by the homogeneous variance assumption. Vague or flat priors such as N(0, 10, 000) are recommended for the basic parameters (7). However, The induced prior on odds ratio (OR), has a big probability on an unrealistic region of odds ratio such that Pr(OR > 1,000) ≈ 0.47 and Pr(OR > 10^29^) ≈ 0.25. However, it provides vague information on the realistic region of the odds ratio and as a result, the posterior distribution depends little on such prior distribution (29). There is no strict rule for selecting a prior for σ^2^. The general practice is to set weakly informative priors, such as σ ~ Unif(0, 2) or σ ~ Unif(0, 5), or non-informative priors such as 1/σ^2^ ~ Gamma(0.001, 0.001). In cases where the data are insufficient, a non-informative prior for σ^2^ would be likely to make the posterior distribution include extremely large or small values (30, 31). Lambert et al. (31) conducted a simulation study using 13 vague priors and found that the use of different vague prior distributions led to markedly different results, particularly in small studies. On the basis of those results, Lambert et al. (31) suggested that in any Bayesian analysis, researchers should assess the sensitivity of the results to the choice of the prior distribution for σ^2^, because “vague” is not the same in all cases. For example, if the prior chosen for the between-study variance is Unif(0,5), a sensitivity analysis for that prior could look at how the posterior estimates of the treatment effects (e.g., the log odds ratios or the absolute risks) in the network meta-analysis would change if the prior is changed to Unif(0,2), Unif(0,10), or some other distribution. If the posterior estimates do not change substantially, the results can be considered insensitive to the choice of prior parameter values. Informative priors can be considered if there are reasonable estimates of σ^2^ available from another, larger network meta-analysis that has the same context and similar treatments as the analysis under construction (7, 32). Having considered the choice of a random effects or fixed effects model, the handling of multi-arm trials, and the choice of priors, the specification of the comparative effects model is complete. Figure 1 illustrates an example of the coding of a comparative effects model in the general_model.bug code.

### 3.5. The Baseline Effects Model–the Log Odds of the Event

After defining the comparative effects model and the priors for the parameters to be estimated, the next step in a Bayesian network meta-analysis is to model the baseline effect. Although it is possible to conduct a Bayesian network meta-analysis without a baseline effects model, the baseline effects model allows for some unique and informative outputs from the analysis. If we are only interested in the estimates of the log odds ratios and the odds ratios, then there is no need to make a baseline effects model. The baseline effects model refers to the distribution of the event for the baseline treatment, that is, the log odds of the event for the baseline treatment. For example, if in one study 40 out of 100 animals in the baseline group experienced the event, the trial-specific log odds of the event would be log(($\frac{40}{100})/\left(\frac{60}{100}\right)$). A different trial would have different log odds of the event; however, the log odds of the event are assumed to arise from the same distribution in all trials. The reason for modeling the distribution of the event risk in the baseline group is to enable absolute effects (i.e., absolute risk) and comparative effects to be estimated on a risk scale rather than on an odds scale. For example, if we know the log odds ratio for all treatments compared with the baseline treatment, then, given the absolute risk for any one treatment, we can know the absolute risk for every treatment. For example, if we have a log odds ratio of 0.9809 for the comparison between treatment A and the baseline treatment, and if the baseline event risk is 0.2 (e.g., 20 out of 100 exposed subjects experienced the event), then we can determine that the absolute risk for treatment A is 0.4, using the formula

$$\begin{array}{l} p=\frac{\text{OR}\times p_{b}}{1-p_{b}+\text{OR}\times p_{b}}, \end{array}$$

where *p*_*b*_ is the absolute risk for the baseline treatment, and *p* is the absolute risk for any non-baseline treatment. The absolute effect of the baseline treatment is often selected for baseline effect modeling, because the baseline treatment is usually the most common treatment in the network meta-analysis, which means that it has the most data available for estimation of the posterior distribution of the log odds of the event. Suppose there are *N*_*b*_ studies that have the baseline arm. Let θ_*i, b*_ (*i* ∈ {1, …, *N*_*b*_}) be the trial-specific baseline effect (log odds of the event) in a trial *i* (i.e., the log odds). We can use the following formulation to model the baseline effect:

$$\begin{array}{l} \theta_{i,b}\sim \text{N}\left(m,\sigma_{m}^{2}\right). \end{array}$$

**Figure 2:**
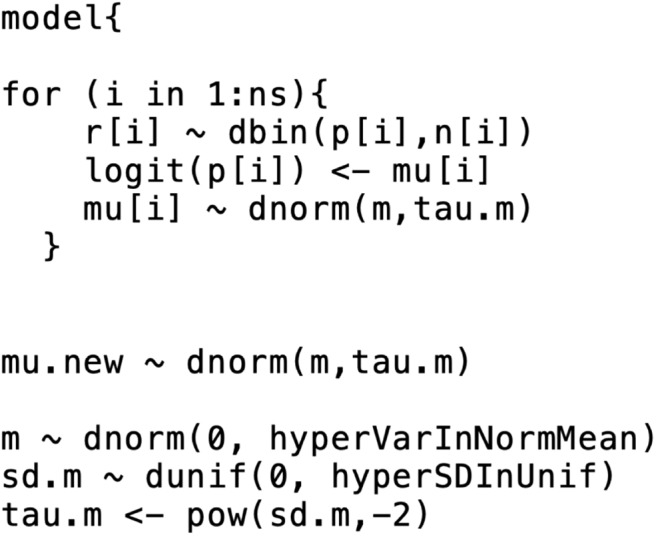
An example of the formatting of the BUGS code for the baseline effects model. This code was modified from code originally published elsewhere (7).

This means that the trial-specific baseline effects come from a normal distribution with mean *m* and variance $\sigma_{m}^{2}$. As with the comparative effects model, we need to select priors for the baseline effects model. The selection of prior distributions for *m* and $\sigma_{m}^{2}$ follows the same considerations as the selection of priors for the effect parameters, that is, the priors should be weakly informative or non-informative [e.g., *m* ~ N(0, 10000), and σ_*m*_ ~ Unif(0, 5)]. From a coding perspective, there are two ways to incorporate a baseline effects model into the comparative effects model. The first approach is to run separate models, beginning with the baseline effects model. The baseline model yields the posterior distribution summaries of *m* and σ_*m*_ (or $\sigma_{m}^{2}$). The posterior means (denoted by $\hat{m},{\hat{\sigma }}_{m}^{2}$) are then inserted into the comparative effects model and the baseline effect can be generated from N$\left(\hat{m},{\hat{\sigma }}_{m}^{2}\right)$ in the comparative effects model. Other quantities of interest (e.g., the absolute risk for the other treatments) can then be estimated. The first approach relies on the assumption that the posterior distribution of the baseline effect is approximately normal. Dias et al. (33) suggests checking that assumption (e.g., with Q-Q plot or Kolmogorov–Smirnov test), although the assumption is usually found to hold. The second approach to incorporate the baseline effects model into the comparative effects model is simultaneous modeling of the baseline effect and the comparative effects. That approach can have a substantial impact on the relative effect estimates, however. For more details on the simultaneous modeling of baseline and comparative effects, refer to Dias et al. (33). Figure 2 shows the incorporation of a baseline effects model, which can be used to obtain *m* and σ_*m*_ (or $\sigma_{m}^{2}$).

## 4. The Workflow for Conducting a Bayesian Network Meta-Analysis

### 4.1. Data Input

The data used in network meta-analyses are typically arranged in one of three formats: one study per row, one comparison per row (contrast-level data), or one arm per row (arm-level data only). In our network meta-analysis functions, we use the one-study-per-row format. The example data that we use in the following analysis are shown in arm-level format in Table 1. In the example data, there are five treatments (A, B, C, D, and E). The baseline treatment is A. It is essential that the baseline treatment is indexed as one (1) and that the data are organized such that the baseline treatment arms are always the “Arm1” treatment. If there are trials with more than two arms, then corresponding columns (e.g., “Number of Events in Arm.1,” “Arm.3,” “Arm3”) can simply be added to the dataset. Table 2 shows the same data arranged in contrast-level format.

**Table 1:** Example data arranged in arm-level format.

**Study**	**Number of event in arm.1**	**Number of event in arm.2**	**Number of event in arm.3**	**Total number in arm.1**	**Total number in arm.2**	**Total number in arm.3**	**Total**	**Arm.1**	**Arm.2**	**Arm.3**	**Number of arms**	**Arm1**	**Arm2**	**Arm3**
1	25	17	20	41	84	100	225	A	B	C	3	1	2	3
2	36	32		41	84		125	A	B		2	1	2	
3	19	7		25	25		50	A	B		2	1	2	
4	20	5		25	50		75	A	B		2	1	2	
5	41	47		50	100		150	A	B		2	1	2	
6	122	69		160	314		474	A	E		2	1	5	
7	236	53		402	399		801	A	E		2	1	5	
8	23	15		27	52		79	A	E		2	1	5	
9	175	166		281	274		555	B	E		2	2	5	
10	57	20		119	118		237	B	E		2	2	5	
11	19	12		100	100		200	B	E		2	2	5	
12	19	7		100	100		200	B	E		2	2	5	
13	16	21		258	254		512	B	E		2	2	5	
14	42	15		50	100		150	A	B		2	1	2	
15	64	34		154	154		308	A	C		2	1	3	
16	34	15		53	106		159	A	C		2	1	3	
17	70	42		130	129		259	A	C		2	1	3	
18	92	31		121	121		242	A	C		2	1	3	
19	35	20		45	90		135	A	C		2	1	3	
20	41	62		59	117		176	A	C		2	1	3	
21	37	15		43	85		128	A	C		2	1	3	
22	16	21		18	35		53	A	C		2	1	3	
23	70	35		122	123		245	A	B		2	1	2	
24	204	71		300	300		600	A	D		2	1	4	
25	111	66		523	526		1049	C	E		2	3	5	
26	60	50		305	297		602	B	C		2	2	3	

The last two columns are the treatment indexes used to distinguish different treatments in the code.

**Table 2:** Example data in contrast-level format.

**Study**	**Arm.1**	**Arm.2**	**Arm.3**	**Number of arms**	**lor 2**	**lor 3**	**se 2**	**se 3**	**Arm1**	**Arm2**	**Arm3**	**V**	**PLA lo**
1	A	B	C	3	−1.82	−1.83	0.42	0.41	1	2	3	0.10	0.45
2	A	B		2	−2.46		0.53		1	2			1.97
3	A	B		2	−2.10		0.65		1	2			1.15
4	A	B		2	−3.58		0.69		1	2			1.39
5	A	B		2	−1.64		0.42		1	2			1.52
6	A	E		2	−2.43		0.23		1	5			1.17
7	A	E		2	−2.23		0.18		1	5			0.35
8	A	E		2	−2.65		0.62		1	5			1.75
9	B	E		2	−0.07		0.17		2	5			
10	B	E		2	−1.51		0.31		2	5			
11	B	E		2	−0.54		0.40		2	5			
12	B	E		2	−1.14		0.47		2	5			
13	B	E		2	0.31		0.34		2	5			
14	A	B		2	−3.39		0.48		1	2			1.66
15	A	C		2	−0.92		0.25		1	3			−0.34
16	A	C		2	−2.38		0.40		1	3			0.58
17	A	C		2	−0.88		0.26		1	3			0.15
18	A	C		2	−2.22		0.30		1	3			1.15
19	A	C		2	−2.51		0.44		1	3			1.25
20	A	C		2	−0.70		0.34		1	3			0.82
21	A	C		2	−3.36		0.52		1	3			1.82
22	A	C		2	−1.67		0.83		1	3			2.08
23	A	B		2	−1.22		0.27		1	2			0.30
24	A	D		2	−1.92		0.18		1	4			0.75
25	C	E		2	−0.63		0.17		3	5			
26	B	C		2	−0.19		0.21		2	3			

“*lor 2” is the column of log odds ratio of “Arm 2” to “Arm 1.” “se 2” shows the corresponding within-trial standard error. The column labeled “V” contains the variance of the log odds of “Arm 1” only if the trial has more than two arms, as discussed in the section “Multi-arm trials.” The column labeled “PLA lo” contains the log odds for the baseline treatment*.

### 4.2. Running the Analysis

After we select studies that meet the transitivity assumption, extract the data and arrange them in the necessary format, decide upon a fixed or random effects model, set the priors for the basic parameters, determine the boundaries of the between-trial variance based on the data, and obtain $\hat{m}$ and ${\hat{\sigma }}_{m}$ (or ${\hat{\sigma }}_{m}^{2}$) from the baseline effects model, the next step is to run the network meta-analysis.

### 4.3. A Description of the Workflow of a Network Meta-Analysis

The workflow of a Bayesian network meta-analysis can be described as follows:

1. Use the comparative effects model and a Markov chain Monte Carlo (MCMC) process to obtain the posterior distributions of the log odds ratios for the basic parameters. From those basic parameters, obtain the posterior distributions of the functional parameters. After running the model the next sub-steps are to:
  - Assess the convergence by evaluating the trace plots and convergence criteria such as the potential scale reduction factor proposed by Gelman and Rubin (34).
  - Check the goodness of the model's fit using the (residual) deviance. It is the posterior mean of the difference in the negative 2 × log likelihood between the current model and the saturated model (35). An empirical rule to check if the model fits well (7) is that the value of the residual deviance should be close to the number of independent data points (36).
  - Obtain the summary information [mean, standard deviation (S*D*)] of the distributions of basic parameters and functional parameters from the comparative effects model and also the summary information (mean, *SD*) of the distributions of basic parameters from the pairwise comparative effects model.
2. Use pairwise comparative effects models and the MCMC process to obtain the posterior distribution of the log odds ratio for the treatments that have direct comparisons that can be used later to check the consistency assumption. This step is essentially a series of Bayesian pairwise meta-analyses based on direct estimates. Hence, no indirect evidence is used in the estimation procedure. After running the model again the next sub-steps are to:
  - Ensure convergence by evaluating the trace plots and convergence criteria.
  - Obtain the summary information of the distributions of basic parameters and functional parameters from the pairwise comparative effects model.
3. Using data from Step 1 and 2, assess the consistency assumption for the treatment comparisons for which there is direct evidence. This is done by subtracting the mean estimated log odds ratios obtained from the posterior distributions of the pairwise meta-analyses from the mean estimated log odds ratios obtained from the posterior distributions of the network meta-analysis and looking for inconsistencies (37). The “indirect estimates” can be obtained by
  $$\begin{array}{l} \;{\hat{d}}_{\text{indir}}=\text{Var}\left({\hat{d}}_{\text{indir}}\right)\left(\frac{{\hat{d}}_{\text{NMA}}}{\text{Var}\left({\hat{d}}_{\text{NMA}}\right)}-\frac{{\hat{d}}_{\text{dir}}}{\text{Var}\left({\hat{d}}_{\text{dir}}\right)}\right),\\ \frac{1}{\text{Var}\left({\hat{d}}_{\text{indir}}\right)}=\frac{1}{\text{Var}\left({\hat{d}}_{\text{NMA}}\right)}-\frac{1}{\text{Var}\left({\hat{d}}_{\text{dir}}\right)}, \end{array}$$
  and should be consistent with the direct estimates. For example, if the pairwise comparison of treatment A with treatment B gives a mean difference in effect size of 1.2, then the indirect comparison of those treatments should give a mean difference in effect size that is positive and of similar magnitude. The hypothesis that the difference between the direct and indirect estimates is zero can be tested using a z-score and corresponding p-value. Such hypothesis tests are often very low powered, however, so it is recommended to also visually evaluate the magnitude and direction of the indirect effects and determine if they are consistent with the direct effects.

If there is no evidence of inconsistency, and residual deviance is also not a concern, then the network meta-analysis is complete. If there is inconsistency, then it is necessary to evaluate the included studies to determine the cause of the inconsistency. In our experience, we once identified an issue with inconsistency that appeared to be linked to a single study that contained results that were not consistent with those of the other studies in the network. In that situation, we removed the problematic study from the network and performed the network meta-analysis without it. More information about that example can be found elsewhere (6).

The next step is to convert the distributional information about the basic and functional parameters into a form that is appropriate for presentation and interpretation. First, we will discuss the estimates of the treatment effects (i.e., the log odds ratios, odds ratios, and risk ratios). Then, we will discuss how to derive information from those estimates. In reality, the distributions of the treatment effects are obtained during the performance of the network meta-analysis. When the MCMC process is conducted, each simulation yields an odds ratio, a baseline event risk, and a risk ratio. The posterior distributions of the parameters and the summary statistics for the distributions are then extracted from the raw data produced by the simulations. Thus, it is possible to report the following:

- All possible log odds ratios with 95% credible intervals as shown in Table 3. These are estimated from the model using the indirect and direct information.
- All possible pairwise odds ratios with 95% credible intervals (Table 4). These are estimated by converting each log odds ratio to an odds ratio during each simulation and then obtaining the posterior distribution of the odds ratios. These cannot be obtained by exponentiation of the mean or the limits of the posterior distribution of the log odds ratio.
- All possible pairwise risk ratios with 95% credible intervals (Table 5). These estimates are obtained for each simulation by using the basic parameters (log odds ratios) and the baseline risk to calculate the probability of an event for each treatment with the expit formula. For example, if for a particular simulation the log odds ratio for treatment B compared with treatment A is 0.9809 (odds ratio of 2.667), and the baseline risk for treatment A is 20%, then the risk of an event for treatment B is 40%. The treatment event risks are then used to create risk ratio estimates (40/20%).

**Table 3:** The estimated log odds ratio from all possible pairwise comparisons in the network meta-analysis of five treatment groups.

**E**	−0.648	−0.689	−0.475	−2.576
(−2.304_0.983)	**D**	−0.041	0.174	−1.928
(−1.394_0.017)	(−1.646_1.559)	**C**	0.214	−1.887
(−1.058_0.108)	(−1.421_1.797)	(−0.422_0.850)	**B**	−2.101
(−3.208_-1.969)	(−3.451_-0.415)	(−2.404_−1.398)	(−2.653_−1.577)	**A**

*All the point estimates are the posterior mean of the log odds ratio of the upper left treatment to the lower right treatment. For example, −2.101 is the posterior mean of the log odds ratio of treatment B to treatment A. (−2.653_−1.577) is the 95% credible interval of the log odds ratio of treatment B to treatment A*.

**Table 4:** The estimated odds ratio from all possible pairwise comparisons in the network meta-analysis of five treatment groups.

**E**	0.743	0.535	0.650	0.080
(0.100_2.672)	**D**	1.347	1.678	0.196
(0.248_1.017)	(0.193_4.753)	**C**	1.305	0.157
(0.347_1.114)	(0.241_6.033)	(0.656_2.341)	**B**	0.127
(0.040_0.140)	(0.032_0.660)	(0.090_0.247)	(0.070_0.207)	**A**

*All the point estimates are the posterior mean of the log odds ratio of the upper left treatment to the lower right treatment. For example, 0.127 is the posterior mean of the odds ratio of treatment B to treatment A*.

**Table 5:** The estimated risk ratio from all possible pairwise comparisons in the network meta-analysis of five treatment groups with the summary of baseline risk to be mean = 0.713, median = 0.728, 2.5% limit = 0.45, 97.5% limit = 0.899.

**E**	0.781	0.616	0.711	0.252
(0.208_2.309)	**D**	1.074	1.260	0.423
(0.326_1.012)	(0.263_2.543)	**C**	1.200	0.411
(0.422_1.083)	(0.310_3.059)	(0.736_1.894)	**B**	0.356
(0.102_0.496)	(0.094_0.900)	(0.205_0.675)	(0.168_0.621)	**A**

*All the point estimates are the posterior mean of the risk ratio of the upper left treatment to the lower right treatment. For example, 0.356 is the posterior mean of the risk ratio of treatment B to treatment A*.

Apart from estimating all possible pairwise treatment effects using direct and indirect data on different scales, it is also possible to create other outputs that help to illustrate aspects of the data. There are many options, but here we discuss only a few. Many outputs are based on the creation of an indicator variable that takes a given value at a frequency proportional to the probability of an event. The indicators can be created during the simulation process or *post-hoc* in R. The code in the Appendix provides examples of both approaches.

**Figure 3:**
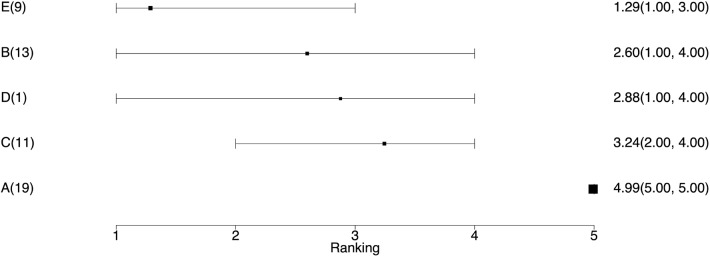
The ranking plot. The left column is the treatment name with the number of studies including that treatment. The right column is the posterior mean ranking of the absolute risk of each treatment and 95% credible interval. Lower rankings have lower incidence of the disease.

- The average ranking of each treatment (Table 6). Once the event probability has been determined for each simulation, it is then possible to rank the event risk across all the treatments. A numerical value ranging from 1 to the total number of treatments is then assigned to each treatment. The researcher can determine what is considered a good or high rank based on the event and what value to assign the most desirable rank. Usually, a rank of 1 is assigned as the preferred result. For example, consider one simulation where the probability of an event for treatments A, B, C, D, and E is 10, 15, 17, 20, and 30%, respectively. If the event is a desirable characteristic, such as a cure, then the treatments A, B, C, D, and E would be assigned the ranks 5, 4, 3, 2, and 1, respectively. In the next simulation, the probability of the event for treatments A, B, C, D, and E might be 5, 22, 17, 24, and 33%, respectively, so treatments A, B, C, D, and E would be ranked as 5, 3, 4, 2, and 1, respectively. In a Bayesian analysis, the posterior samples from all three chains can be used to create a posterior distribution of the rankings. The summary statistics of the posterior distribution of the rankings can be reported. Often the mean or median of the posterior distribution of the rankings and the 95% credible intervals of the rankings are used to create a ranking plot, as shown in Figure 3.
- The probability of being the best (or worst) treatment (Table 7). Using the data from the rankings, it is possible to sum the number of times each treatment received the highest (or lowest) rank. The sum can then be reported as the probability that the treatment has the highest (or lowest) rank, which is colloquially interpreted as the probability of being the best (or worst) treatment.
- All possible pairwise comparisons of the probability of being better (Table 8). Using the ranking data, which are based on the event risk data for each treatment, it is possible to sum the proportion of times that one treatment is ranked higher (or has a higher event rate) than another treatment. This can be done using either the ranking data or the event risk data, which both give the same result. In our example data, the probability that B, C, D, and E were better than A was 10%, whereas the probability that B was better than C was 50%.

**Table 6:** Summary of the distribution of the rankings for the five treatments.

**Treatment**	**Mean**	***SD***	**2.5%**	**50%**	**97.5%**
A	4.99	0.09	5	5	5
C	3.25	0.74	2	3	4
D	2.86	1.21	1	3	4
B	2.60	0.75	1	3	4
E	1.29	0.54	1	1	3

**Table 7:** The probability of being the best treatment and the probability of being the worst treatment.

**Treatment**	**Probability of being best**	**Probability of being worst**
A	0.000	0.992
B	0.033	0.000
C	0.015	0.000
D	0.201	0.008
E	0.751	0.000

**Table 8:** The probability that one treatment is better than another, i.e., has lower disease incidence during the study period.

**A**	0.000	0.000	0.008	0.000
1.000	**B**	0.757	0.587	0.052
1.000	0.243	**C**	0.476	0.028
0.992	0.413	0.524	**D**	0.206
1.000	0.948	0.972	0.794	**E**

*The upper quadrant provides the probability that the row treatment is better than the column. For example, there is probability of zero that “A” (1st row) is better than “B” (2nd column) and a probability of 0 that “A” is better than “E”*.

### 4.4. Plots Commonly Used to Show the Results of a Network Meta-Analysis

There are various types of plots that can be used to present the results of a network meta-analysis. Examples of three of the most common types are shown below.

**Figure 4:**
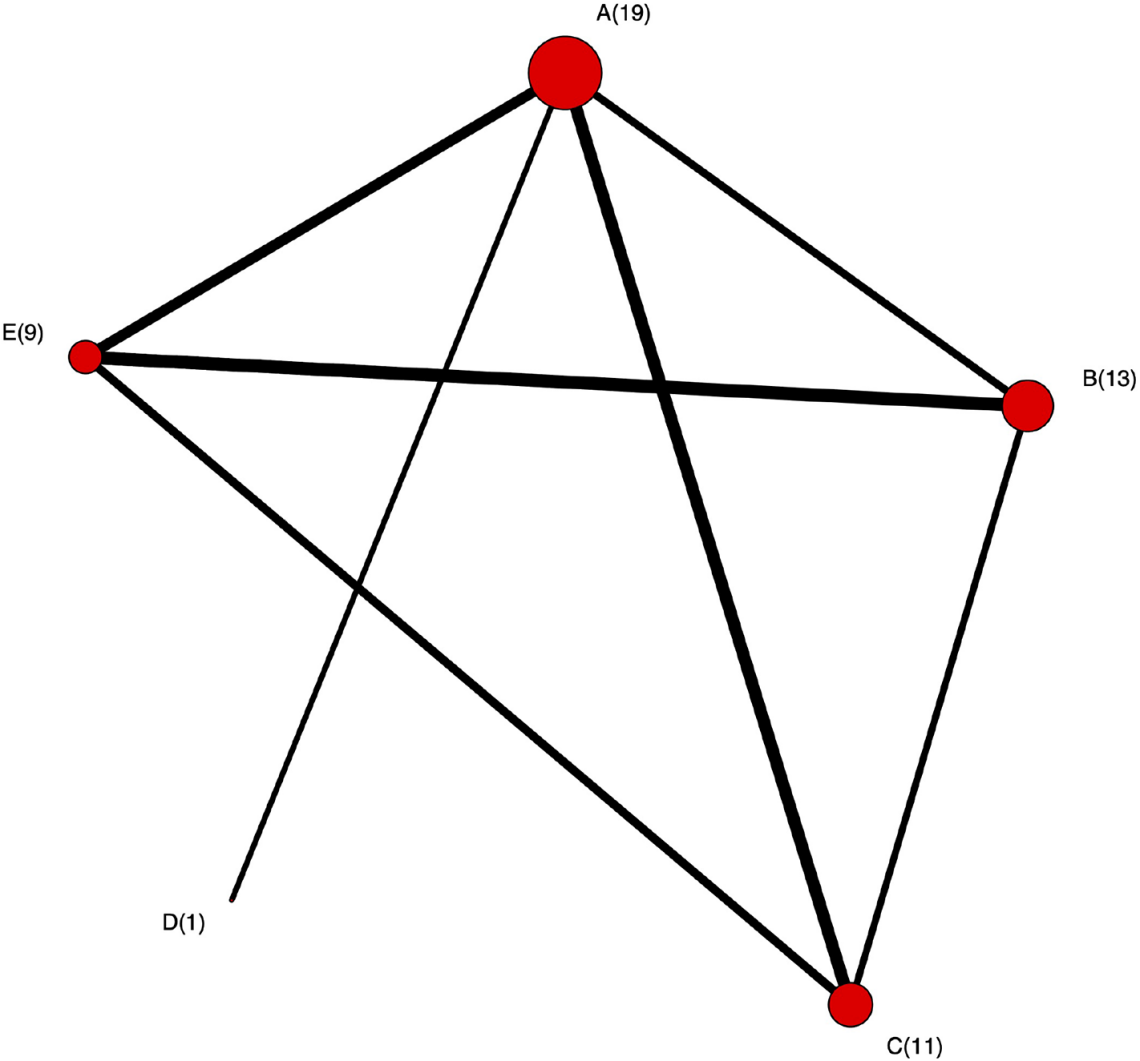
The network plot. Each node represents treatment and the number is the corresponding number of studies including that treatment. An edge between two nodes (treatments) means there were studies comparing these two treatments.

**Figure 5:**
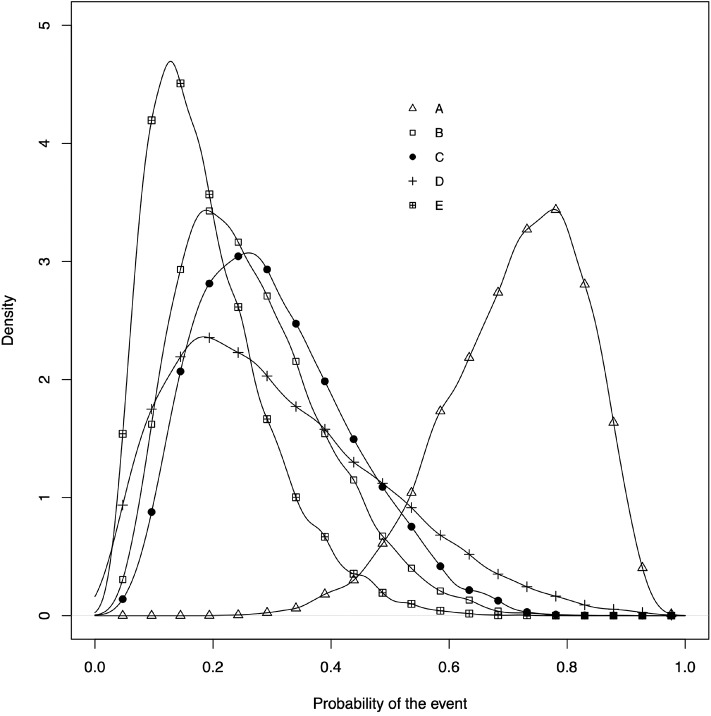
The posterior distribution of the event risk of each treatment.

- The network plot as shown in Figure 4. This plot is a visual representation of the network of evidence. Although we did not discuss the network plot until the end of the tutorial, because it is not technically part of the network meta-analysis, this plot should actually be generated before the network meta-analysis is undertaken. The code provided in the Appendix illustrates how to create the network plot using packages from R. There are also other approaches that can be used to create the network plot. The code in the Appendix includes some common metrics used to describe networks, which are not discussed further here (38).
- The posterior distribution of the event risk (Figure 5). This plot illustrates the posterior distribution of the event risk for each treatment using all posterior samples of that risk.
- The ranking plot (Figure 3). The ranking plot uses the data from the posterior distribution of the rankings to create a forest plot-like graphic using the means and 95% credible intervals of the rankings.

## 5. Discussion

In this tutorial, we described the conceptual framework for a network meta-analysis, explained the step-wise workflow for conducting a network meta-analysis, and provided code in the Appendix that illustrates the mechanics of conducting a Bayesian network meta-analysis. The Bayesian inference tool used in this tutorial is JAGS. Stan Development Team (39), as an alternative Bayesian inference instrument, could also be used to conduct network-meta analysis. As we mentioned in the introduction section, other packages for network meta-analysis like *gemtc* and *BUGSnet* are also available. Compared with *gemtc*, the outputs in our code are more flexible and are shown in table format. Our code can also deal with arm-level data as well as contrast-level data in comparison to *BUGSnet*. Despite these advantages, there are some limitations. Our code focuses on the binary outcome. *gemtc* and *BUGSnet* provide functions handling other types of outcome like continuous and count outcomes.

Network meta-analysis, as a popular method of simultaneously comparing multiple treatments, still presents challenges since it not only has the challenges as in a standard pairwise meta-analysis but also increases the complexity due to the network structure (40). Therefore, some assumptions are made to ensure the validness of a network meta-analysis. The transitivity assumption is that studies can be combined only when they are clinically and methodologically similar (41, 42). This means according to the Cochrane Handbook of Systematic Reviews “that different sets of randomized trials are similar, on average, in all important factors other than the intervention comparison being made” (43). For example, the distributions of effect modifiers should be similar across studies (44). Practically, in our BRD example, the transitivity assumption means that each study population would have been eligible for any of the other studies and all study populations were eligible for all treatments. An example of a situation that would violate this transitivity assumption would be a comparison of antibiotic treatment efficacy where one group of trials assessed the response to 1st treatment and another group of trials assessed the treatment response of cattle with a 1st treatment failure (re-pull). Obviously, the cattle in the 1st treatment response are not eligible for the 1st treatment failure studies. The validity of indirect and combined estimates of relative effects would be threatened if this assumption is violated (43). Consistency assumption is a manifestation of transitivity. As we discussed in section 3.2, it requires that the indirect evidence must be consistent with direct evidence. Violation of the consistency assumption would result in inconsistency (45). Although inconsistency model have been proposed to mitigate the violation of this assumption in some way, one still should be cautious when combining studies and choosing which model to use. This tutorial focuses on the statistical aspect of conducting a network meta-analysis while aspects such as defining the research question, searching for studies and assessing the risk of bias within each study (46, 47) are not in the scope of this tutorial.

For readers that are interested in running a simple network meta-analysis without going into any detailed explanation of the underlying process, we believe that the instructions that come with any one of the ever-growing number of software packages for network meta-analysis will provide sufficient information for a successful analysis to be conducted (12, 15–17). More details about interpreting the results of a network meta-analysis can be found on this paper (48).

## Data Availability Statement

The datasets generated for this study can be found in the GitHub [https://github.com/a-oconnor/NETWORK_MA_FRONTIERS_TUTORIAL].

## Author Contributions

DH prepared the draft of the manuscript, wrote the code used to conduct the data analysis, and worked with AO'C to ensure that the interpretation was correct. AO'C prepared the draft of the manuscript, coordinated the project team, assisted with the data analysis, and interpreted the procedure and results of the analysis. CW provided guidance on the conduct of the analysis and commented on the draft of the manuscript to ensure that the interpretation of the analysis was clear. JS provided feedback on the draft to ensure that the interpretation of the analysis was clear. CBW provided feedback on the draft to ensure that the interpretation of the analysis was clear.

## Conflict of Interest

The authors declare that the research was conducted in the absence of any commercial or financial relationships that could be construed as a potential conflict of interest.

## Appendix

The tutorial R project with instructions, data set, scripts, bugs are available at https://github.com/a-oconnor/NETWORK_MA_FRONTIERS_TUTORIAL.
